# Military genomics: a perspective on the successes and challenges of genomic medicine in the Armed Services

**DOI:** 10.1002/mgg3.335

**Published:** 2017-09-14

**Authors:** Mauricio J. De Castro, Clesson E. Turner

**Affiliations:** ^1^ United States Air Force Medical Genetics Center 81st Medical Group Keesler AFB Mississippi 39534; ^2^ Division of Genetics Department of Pediatrics Walter Reed National Military Medical Center Bethesda Maryland 20889

## Abstract

We describe the impact genomics has on the health and readiness of the military service member, highlight several examples of the current and future plans for genomic medicine within the military, discuss challenges to implementation and provide recommendations to address some of those challenges.

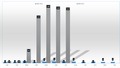

## Introduction

Recent personnel, infrastructure, and technological developments within aspects of the Military Health System (MHS) herald a major entry of the Department of Defense (DoD) into the era of genomic medicine. In this invited commentary, we describe the impact genomics has on the health and readiness of the military service member, highlight several examples of the current and future plans for genomic medicine within the MHS, discuss challenges to implementation and provide recommendations to address some of those challenges.

## Genomic Medicine and the Service Member

Genetics and genomics have been part of military service, albeit a small one, for the better part of half a century (Webber and Witkop 2014). Mirroring the dramatic growth of genetic testing over the last decade in the civilian population (Rubinstein et al. 2013), genomic medicine also is increasingly common in the military healthcare system. Over the last 3 years, we have seen a marked increase in the utilization of genetics and genomics into everyday clinical practice. The mainstreaming of genetics has driven much of this increased interest.

**Figure 1:**
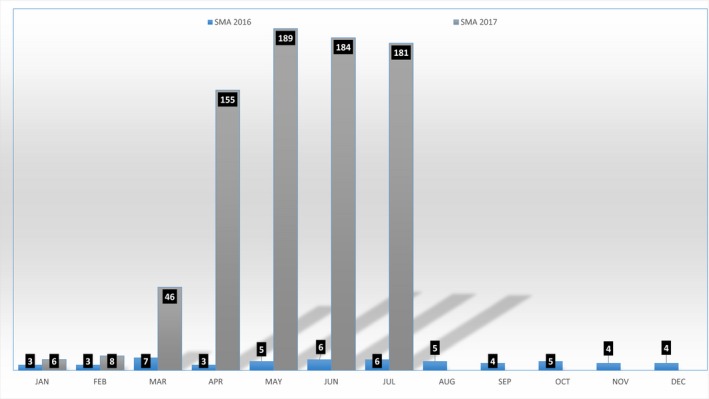
Spinal muscular atrophy (SMA) prenatal screening trend in the DoD from 2016 to July 2017.

As an example, the recently released “Carrier Screening for Genetic Conditions” guidelines from the American College of Obstetricians and Gynecologists (ACOG) recommend that screening for spinal muscular atrophy (SMA) should be offered to all pregnant women (ACOG, 2017). Internal data from the Air Force Medical Genetics Center (AFMGC), the reference molecular diagnostic laboratory for the DoD, showed that military practitioners have quickly begun to incorporate this guideline into their clinical practice (Fig. 1). A similar trend was observed when comprehensive *BRCA1* and *BRCA2* testing was made available through the AFMGC, although reduced cost and increased availability may have contributed as well. These examples highlight the broad consensus that system‐based improvements, such as the issuance of evidenced‐based guidelines and improved access to genetic testing, can in the right circumstances, lead to appropriate utilization of these technologies (Lieberman et al. 2017).

This increase in the utilization of genetic services has been in part driven by their popularization with the general public (Jolie 2013), this in turn has led to increased interest from our patients in inherited conditions and genetic testing. By far, most of the military medical beneficiary population are young, healthy individuals, and so, most genetic testing performed within the MHS is predictive genetic testing (e.g., family history of cancer) and carrier screening. All of these patients should ideally receive pre and posttest counseling with their testing (Stanislaw et al. 2016). As a result, there has been a steady increase in the number of referrals for genetic services within Military Treatment facilities (MTFs), with wait times to see a geneticist in excess of 6 months and longer.

The increasing role that genetics plays in the practice of medicine has elicited interest by military leadership in understanding the effect these changes in provider practices will have on service members. Collecting such information has proven to be a difficult task as the military healthcare system is a massive enterprise with hundreds of thousands of beneficiaries spread throughout the world, sometimes in very austere environments. In contrast, the military genetics community is small compared to other specialties. There are only a handful of active duty geneticists and as many genetic counselors. Current efforts are underway to better address some of these questions.

## Current Initiatives

In the face of the above challenges, and in the spirit of partnership fostered by the Precision Medicine Initiative and the Cancer Moonshot (White House, 2016), multiple collaborations between the Services, with other federal entities and with civilian partners, have sought to answer some of the difficult questions regarding implementation of genomic medicine within the MHS. We highlight several of these ongoing collaborations as examples of the ways in which the DoD is poised to provide major contributions to genomic medicine.

The MilSeq Project is an Air Force Medical Support Agency (AFMSA)‐funded collaborative effort with Harvard Medical School and the Baylor School of Medicine that seeks to answer questions regarding the integration of genomic medicine into the day‐to‐day practice of medicine. This project aims to better define Active Duty provider and patient attitudes regarding the integration of extensive genomic information in their medical records and what impact, if any, this will have on a service member. As an important side note, the MilSeq Project will also pilot the provision of genomic education to nongenetics providers that opt to be part of the study.

A second initiative, the American Genome Center (TAGC), is a high throughput genome sequencing center established last year at the Uniformed Services University of the Health Sciences (USU). TAGC provides core support for genome sequencing, RNA sequencing, and expert bioinformatics analysis to researchers across the DoD and other federal entities. In conjunction with these efforts, a clinical genomics program is being established at USU to support the clinical translation of results obtained from research sequencing at TAGC. This clinical genomics program will also examine the ethical, legal, and social implications of integrating genomic information into the MHS in an effort to contribute evidence to guide future DoD policy decisions.

The DoD is also participating in the Applied Proteogenomics OrganizationaL Learning and Outcomes (APOLLO) Network is a federal partnership leveraging the scientific and technical capabilities of the National Cancer Institute (NCI), the DoD and Veterans Affairs (VA). APOLLO will combine data from genomic and proteogenomic analyses of germline and somatic tissue from cancer patients in order to inform individualized medical management. Through The Murtha Cancer Center at Walter Reed National Military Medical Center and USU the DoD will be contributing state‐of‐the‐art biobanking capabilities. TAGC will be performing genome and RNA sequencing of tumor and germline specimens submitted by APOLLO researchers (Fiore et al. 2017).

Lastly, in an effort to advance the development of an adequate evidence base for genetic testing to improve patient care and treatment, the DoD Office of Health Affairs recently asked the National Academies of Sciences, Engineering and Medicine to convene a committee to examine all the currently available evidence and provide recommendations (National Academies, 2017).

The scale of these efforts underscores the important challenge of integrating the data generated from these initiatives into the everyday practice of medicine, a challenge mirrored in the broader genomics community as a whole. We believe that the most important component of all these endeavors will be its appropriate, efficient and successful translation into tangible clinical benefits for our military population, particularly as it impacts military readiness. Critical to that implementation will be provider education and the expansion of genetic counseling services.

The military health care system is an expansive institution with medical treatment facilities (MTFs) and beneficiaries all over the world who move frequently. Our approach to tackling this problem has to be nimble and flexible, it is not currently possible to provide access to a geneticist at every MTF in the DoD. One approach to increase access to expert genetic consultation is telemedicine. In fall 2017, we will start a pilot program that leverages the presence of clinical geneticists and genetic counselors at two MTFs to provide genetic services to military bases within and outside of the continental United States. This pilot program will hopefully lead to the development of a centralized genetic service core staffed by clinical geneticists and genetic counselors that will be able to provide genetic counseling, clinical genetic evaluation and medical management recommendations in selected cases. The Veteran's Administration excellent genomic medicine service (telegenomics program) provides the paradigm for what we envision for the DoD.

Studies in the civilian population have reported low confidence on the part of providers in their ability to use genomic data in the care of patients (Korf et al. 2014). The military is no different. Internal data gathered from thousands of commonly ordered genetics tests submitted to the AFMGC suggests a significant proportion of providers have difficulty appropriately understanding and utilizing genetic testing; other data suggest that in some cases, providers have issues interpreting and utilizing the results of said testing. Any future research projects should take this into account by including an educational component for involved providers. Additionally, training the upcoming generations of our military providers is integral to successful long‐term implementation of genomic medicine. Small, pilot studies are underway exploring educational initiatives geared toward Graduate Medical Education and the early stages of training for physician assistants and nurse practitioners.

Finally, in 2014 the Defense Health agency (DHA) sponsored an ongoing demonstration project to expand the pool of non‐FDA approved laboratory developed tests, which includes most clinical genetic testing, that would be covered for TRICARE beneficiaries. This has allowed TRICARE patients access to appropriate genetic testing that was previously denied.

## Challenges Remain

Various challenges remain unaddressed. Within the next few years we expect to have research genome/exome data on thousands of Service Members, with a significant proportion of these individuals receiving reports on CLIA‐certified results. This will include not only primary findings related to the original research or clinical indication for testing, but also information on secondary findings, including disease associated risk alleles, pharmacogenomics and carrier status. We expect to face challenges related to variant interpretation and clinical application of this information, particularly if it relates to a service member's fitness for military service. The issue of fitness for duty is a unique challenge, not usually encountered outside the military.

As in the genomics community as a whole, our main challenge continues to be the dearth of clinical resources. There is a small number of genetic services providers in the Services, and, like the civilian community, the number of interested candidates remains small compared to other medical specialties. Hiring genetic counselors within the DoD has traditionally been difficult, but changes in policies over the last few years have made this process easier.

While some policy issues (De Castro et al. 2015) have been addressed; other issues still remain. The current lack of a comprehensive data sharing policy regarding genomic information within the DoD limits the ability of the MHS to fully realize the clinical benefits of genomic research. Also, research into the ethical, social and behavioral utilization of genomic information, as applied to the military population, is limited. Indeed, understanding these issues is necessary to mitigate the potential negative impacts of these technologies.

On the policy front, DHA has taken steps to address some of these challenges. The MHS Precision Care and Genomic Medicine Advisory panel (PCAP) was created to provide policy, scientific and operational recommendations related to emerging issues in the genomics, multi‐omics, and precision medicine realms. The committee is composed of members of all the Services, DHA, Health and Human Services and the Veterans Health administration among others. In practice, the committee serves as a useful forum for all concerned stakeholders to provide awareness of ongoing initiatives, encourage information sharing, reduce duplication of efforts and enhance collaboration between the Services.

## Conclusion

We are of the opinion that advances in genetics and genomics will continue to transform the everyday practice of medicine. While facing many of the same challenges as other health systems in integrating genomic medicine, the MHS and its beneficiaries face some specific challenges related to the unique aspects of military service. The efforts highlighted here begin the process of implementing genomic medicine within the MHS in order to improve the health of service members and their families.

## Conflict of Interest

None declared.
